# Scorpion Venom Heat-Resistant Peptide Protects Transgenic *Caenorhabditis elegans* from β-Amyloid Toxicity

**DOI:** 10.3389/fphar.2016.00227

**Published:** 2016-07-26

**Authors:** Xiao-Gang Zhang, Xi Wang, Ting-Ting Zhou, Xue-Fei Wu, Yan Peng, Wan-Qin Zhang, Shao Li, Jie Zhao

**Affiliations:** ^1^Department of Physiology, Dalian Medical UniversityDalian, China; ^2^Department of Neurology, the First Affiliated Hospital of Dalian Medical UniversityDalian, China; ^3^Liaoning Engineering Technology Centre of Target-based Nature Products for Prevention and Treatment of Ageing-related NeurodegenerationDalian, China

**Keywords:** scorpion venom heat-resistant peptide, *C. elegans*, alzheimer’s disease, amyloid beta-peptide, behavior

## Abstract

Scorpion venom heat-resistant peptide (SVHRP) is a component purified from Buthus martensii Karsch scorpion venom. Our previous studies found SVHRP could enhance neurogenesis and inhibit microglia-mediated neuroinflammation *in vivo*. Here, we use the transgenic CL4176, CL2006, and CL2355 strains of *Caenorhabditis elegans* which express the human Aβ_1-42_ to investigate the effects and the possible mechanisms of SVHRP mediated protection against Aβ toxicity *in vivo*. The results showed that SVHRP-fed worms displayed remarkably decreased paralysis, less abundant toxic Aβ oligomers, reduced Aβ plaque deposition with respect to untreated animals. SVHRP also suppressed neuronal Aβ expression-induced defects in chemotaxis behavior and attenuated levels of ROS in the transgenic *C. elegans*. Taken together, these results suggest SVHRP could protect against Aβ-induced toxicity in *C. elegans*. Further studies need to be conducted in murine models and humans to analyze the effectiveness of the peptide.

## Introduction

Alzheimer’s disease (AD) is the most common progressive neurodegenerative disease (Goedert and Spillantini, 2006), affecting a large proportion of elderly individuals in the world (Sloane et al., 2002). More recently, numerous studies have suggested that soluble, small oligomeric forms of amyloid beta-peptide (Aβ) are the main toxic species to neurons (Kayed et al., 2003; Nimmrich and Ebert, 2009; Taylor et al., 2010). Accordingly, the focus of the research into molecules able to delay AD occurrence and to relieve its symptoms has shifted from hindering fibril growth to avoiding the appearance of toxic oligomeric intermediates.

Transgenic *Caenorhabditis elegans* (*C. elegans*) which express the human Aβ_1-42_ offer an original *in vivo* model for investigating the toxicity specifically related to small oligomeric Aβ (Link, 1995). Transgenic *C. elegans* strain, CL4176, expressing human Aβ_1-42_ in muscle cells under a temperature-inducible system (Link et al., 2003), become rapidly paralyzed. Another *C. elegans* strain, CL2006, which constitutively expresses human Aβ_1-42_ in body wall muscle tissues (Link, 1995, 2005), displays an age-related progressive reduction of muscle specific motility which is related to the accumulation of both Aβ_1-42_ fibrils and oligomers. Two characteristic neuronal controlled behaviors, 5-HT sensitivity and chemotaxis, are defects in CL2355 which has inducible neuronal Aβ expression (Wu et al., 2006). All of these strains have already been used to screen for drugs aimed to ameliorate Aβ toxicity such as Gingko biloba extract EGb 761(Wu et al., 2006), green tea component epigallocatechin gallate (Abbas and Wink, 2010) and other compounds (McColl et al., 2009; Morita et al., 2009; Dostal et al., 2010; Yu et al., 2010; Martorell et al., 2013).

Many natural product-based therapies have been used to prevent or treat age-related deterioration in cognitive or memory function (Howes and Houghton, 2003; Jiang et al., 2003; Yan et al., 2007). Buthus martensii Karsch (BmK) and its venom have been used as drugs for treating nervous system diseases in traditional Chinese medicine, such as epilepsy and cerebral infarction (Wang et al., 2009) for thousands of years. Obviously, at first glance, it may seem odd to consider scorpion toxins as potential drugs. However, scorpion venoms are a complex mixture of about 100 to 1000 different components. Typically, low molecular mass (<3 kDa) peptides derived from scorpion venoms are highly diverse in both their primary structures and biological activities and this renders them potentially intriguing therapeutic agents to explore (Keramidas et al., 2004; Zeng et al., 2005; Elgar et al., 2006).

Our laboratory isolated one scorpion venom heat-resistant peptide (SVHRP) from BmK venom (Wang et al., 2014; Yin et al., 2014; Cao et al., 2015). Our previous studies showed that this peptide had neurotrophic and neuroprotective effects (Yin et al., 2014). More recently, it was observed that SVHRP could enhance neurogenesis and stimulate neural maturation by up-regulating Brain Derived Neurotrophic Factor (BDNF) both *in vivo* and *in vitro* (Wang et al., 2014). In addition, previous studies have observed that Buthus martensi Karsch extracts exert anti-inflammatory effects related to the inhibition of neutrophil functions and of NO and PGE2 production through the transcription factors NF-(kappa) B and AP-1(Kim et al., 2005). Neuronal death is considered as main cause of dementias and neuroinflammation play an important role in the progression of AD (Calsolaro and Edison, 2016). Taken together, these results suggest us SVHRP may be effective in AD therapy.

In this study, we used transgenic *C. elegans* that exhibit several pathological behaviors induced by Aβ toxicity as a simplified invertebrate model of AD (Link, 1995, 2005), to evaluate the protective effects of SVHRP against Aβ toxicity *in vivo* and elucidate some of the mechanisms involved in protective effects of SVHRP in *C. elegans*.

## Materials and Methods

### Isolation of Scorpion Venom Heat-Resistant Peptide

The isolation of SVHRP from BmK venom has been reported in our previously published paper (Cao et al., 2015). All drugs for treatment of experimental animals were added directly to the food.

### *Caenorhabditis elegans* Strain

The wild-type *C. elegans* strain N2, the transgenic strain CL2006, the transgenic strain CL2355 and the control strain CL2122, transgenic strain CL4176 and the control CL802 strain were used. The CL2006 strain constitutively expresses Aβ_1-42_ in the cytoplasm of body wall muscle cells, while the transgenic CL4176 worms express muscle-specific Aβ_1-42_ by raising the temperature from 16 to 23°C. The CL2355 strain expresses Aβ_1-42_ in the neuronal cells and the expression of neuron-specific Aβ_1-42_ depends on temperature upshift. All of the *C. elegans* strains were purchased from *Caenorhabditis* Genetic Center (University of Minnesota, USA).

### *C. elegans* Maintenance and Treatment

The transgenic CL2006 and the wild-type (N2) were propagated at 20°C, while CL2355, CL4176, and their controls were maintained at 16°C. All *C. elegans* strains were routinely propagated on solid nematode growth medium (NGM) with 100 μl spots of OP50 (*Escherichia coli* strain) as a food source. To prepare age-synchronized nematodes, worms were allowed to lay eggs for 4–6 h (overnight for Western blotting of CL2006 and CL4176). Then, isolated the adult worms from the synchronized eggs (day 1) and the synchronized eggs were cultured on fresh NGM plates (containing vehicle or SVHRP) in either 20°C or a 16°C (for CL4176, CL2355, and their control strains). In the experiments, the worms were fed with the drugs from the egg through to adult stages and transferred to new plates (containing different concentrations of drugs) every day.

### Paralysis Asssays

Synchronized eggs of CL802 and CL4176 were maintained at 16°C, on the NGM plates containing vehicle or different concentrations of drugs. Transgene expression was induced by upshifting the temperature from 16 to 23°C, started at the 36th hour after egg laying and lasted until the end of the paralysis assay. The scoring was performed at an hourly interval typically after 24 h at 23°C until the last worm became paralyzed. The worms that did not move or only moved their head when gently touched with a platinum loop were scored as paralyzed. Each experiment was performed using at least 100 worms.

### Western Blotting of Aβ Species

The Aβ species in the transgenic *C. elegans* strains was identified by immunoblotting using a Tris-Tricine gel and the standard Western blotting protocol except that the polyvinylidene difluoride membranes were boiled for 5 min after the transfer. After the experimental treatments, the worms were collected, washed with M9 buffer, flash frozen in liquid nitrogen, sonicated in the lysis buffer (62 mM Tris-HCl pH 6.8, 5% β-mercaptoethanol (v/v), 10% glycerol (v/v), 2% SDS (w/v), and 1X protease inhibitor cocktail), and heated with sample buffer containing 5% β-mercaptoethanol. After heating with the sample buffer, the proteins were cooled and equal amounts of the total protein (60-80 μg) were loaded in each lane. Amyloid protein species were detected with 6E10 monoclonal antibody (1:750, Covance); secondary anti-mouse IgG peroxidase conjugate (1:5000; Sigma). Tubulin was detected with anti-tubulin antibody (1:2000, Abcam). The mean densities of β-amyloid reactive bands were analyzed by ImageJ (National Institutes of Health, USA).

### Fluorescent Staining of β-Amyloid with Thioflavin-S

Age-synchronized transgenic CL2006 worms fed or not fed with SVRHP were incubated at 20°C and at 120 h of age the worms were fixed overnight in 4% paraformaldehyde/phosphate-buffered saline (PBS), pH 7.4, at 4°C. Then the nematodes were permeabilized in 1% Triton X-100, 5% fresh β-mercaptoethanol, 125 mM Tris, pH 7.4, in a 37°C incubator for another 24 h. The worms were stained with 0.125% thioflavin S (Sigma) in 50% ethanol for 2 min. The samples were then destained with sequential washes with ethanol, mounted on slides for microscopy and observed with a microscope (DM4000B; Leica, Germany) equipped with a digital camera. Fluorescence images were acquired using a CDD. The number of thioflavin S positive spots in the head region of worms fed with vehicle (*n* = 72) or SVHRP (*n* = 72) were scored. The data expressed as Aβ deposits/anterior area.

### Measurement of Reactive Oxygen Species (ROS) in *C. elegans*

Intracellular ROS was measured in *C. elegans* using 2, 7-dichlorofluorescein diacetate (H2DCF-DA) as described previously (Kampkotter et al., 2007). Briefly, age-synchronized C. *elegans* (50 eggs per plate) were transferred to NGM plates containing vehicle or different concentrations of SVHRP (2, 20, 40 μg/ml) and incubated for 36 h at 16°C. The worms were collected into a microfuge tube at 36 h after temperature upshift, washed twice with PBS to remove *E. coli* (OP50) and transferred into the wells of 96-well plate containing 200 μl of PBS, 0.01% Tween-20 and 50 Mm H2DCF-DA (final concentration in PBS). The fluorescence was quantified in a Synergy HT microplate fluorescence reader (Bio-Tek Instruments, Winooski, VT, USA) every 30 min for 2.5 h at 37°C using the excitation at 485 nm and emission at 530 nm. The fluorescent images of DCF were taken with a FITC excitation and emission filter setup.

### Chemotaxis Assays

Chemotaxis assays (see **Figure 5A**) were performed as described by Olivia Margie (Margie et al., 2013). Synchronized transgenic *C. elegans* CL2355 and its control strain CL2122 were treated with or without SVHRP, starting from the egg. They were cultured in 16°C for 36 h, and then in 23°C for another 36 h. The worms were then collected and assayed in 100 mm plates containing 25 mM phosphate buffer, pH 6.0, 1 mM MgSO_4_, 1 mM CaCl_2_, and 1.9% agar. 1 μl of 0.25M sodium azide along with 1 μl of odorant (0.1% benzaldehyde in 100% ethanol) were added to the “attractant” spot. On the opposite side of the attractant spot, 1 μl of control odorant (100% ethanol) and 1 μl drop of sodium azide were added. Immediately after, pipette 2 μl of the worms (about 60 worms) to the center of the plate. Incubate the assay plates at 23°C for 1 h and then the number of worms in each quadrant was scored. Calculate the Chemotaxis index (CI) using Equation: CI = (number of worms in both attractant quadrants – number of worms in both control quadrants)/total number of scored worms.

### Statistical Analyses

Statistical analysis was performed using *GraphPad* (Version 5.0 for Macintosh OSX, *GraphPad* Software, San Diego, CA, USA). Significance was assessed by a one-way analysis of variance (ANOVA), followed by Newman–Keuls’s Test, or Student’s *t*-test of two groups. Values of *p* < 0.05 were considered statistically significant.

## Result

### Aβ-Induced Paralysis is Alleviated by SVHRP in Transgenic *C. elegans*

**FIGURE 1:**
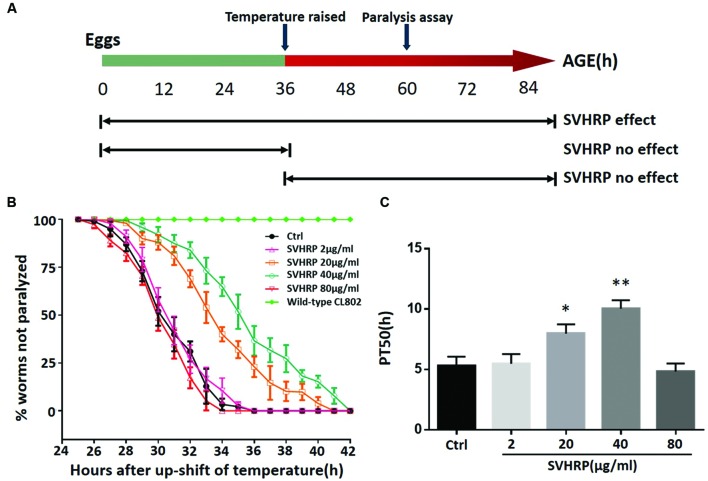
**Scorpion venom heat-resistant peptide (SVHRP) delayed β-amyloid induced paralysis in transgenic *Caenorhabditis elegans* strain CL4176. (A)** Diagram illustrating the paralysis assays showing the time at which the temperature was raised in CL4176 and CL802 worms, when the paralysis assay was scored and SVHRP protective effect at different treatment regimens. **(B)** Time course of Aβ-induced paralysis in the transgenic CL4176 strain treated with a vehicle (Ctrl) or different concentrations of SVHRP (2-80 μg/ml). Data are shown as percentages ± SD of worms not paralyzed (*n* = 100, three independent assays). **(C)** The paralysis assays were quantified for mean time duration at which 50% worms were paralyzed (PT_50_) from the transgenic worms fed with or without SVHRP. Error bars indicate SD (*n* = 100, three independent assays, ^∗^*p* < 0.05, ^∗∗^*p* < 0.01 vs. control group).

We first investigated whether SVHRP protect against Aβ-induced toxicity in the transgenic *C. elegans* strain CL4176. **Figure 1A** showed when the SVHRP were administered, the time at which the temperature was raised in CL4176 and CL802 worms, and when the paralysis assay was rated. The feeding of CL4176 worms with SVHRP (2, 80 μg/ml) starting at eggs did not delay Aβ-induced paralysis but in the transgenic worms fed with SVHRP (20, 40 μg/ml), a notable delay of paralysis was observed compared with the vehicle-treated controls (**Figure 1B**). For quantitative analysis, we define PT_50_ value, i.e., the time interval, from the onset of paralysis, at which half the worms were paralyzed. The PT_50_ was significantly higher in worms fed the SVHRP (20, 40 μg/ml) compared to the control (**Figure 1C**, 5.3 ± 0.75 for vehicle-treated CL4176 worms and 8.0 ± 0.76, 10.0 ± 0.7 for nematodes fed 20, 40 μg/ml SVHRP). These observations suggest that SVHRP may have the potential to protect against Aβ-induced toxicity in the whole animal.

### SVHRP Affects Aβ Oligomerization in Transgenic *C. elegans*

**FIGURE 2:**
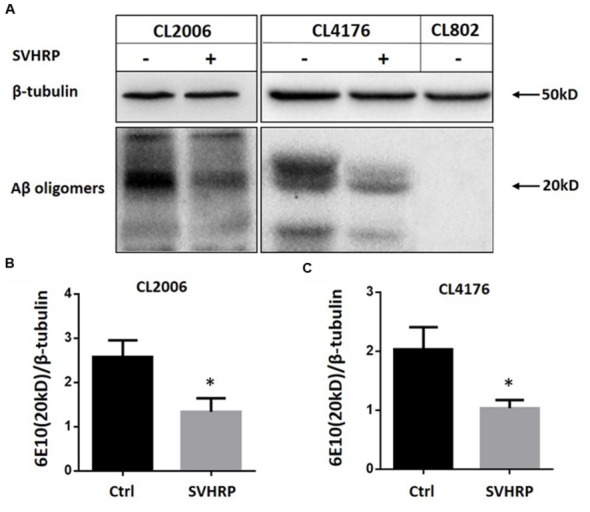
**Scorpion venom heat-resistant peptide reduced β-amyloid oligomers in *C. elegans*. (A)** Representative western blot of Aβ species in CL2006, CL4176, and CL802 worms fed vehicle or SVHRP. CL2006 maintained at 20°C fed with a vehicle (Ctrl), SVHRP (40 μg/ml) for 96 h. CL4176 and CL802 worms fed with a vehicle (Ctrl), SVHRP (40 μg/ml) were maintained for 36 h at 16°C, then the temperature was raised from 16 to 23°C. 25 h later, the worms were collected and equal amounts of protein were loaded in each lane and immunoblotted with anti-Aβ antibody (6E10) or tubulin. Arrows indicate the Aβ oligomers (20 kDa). Quantification of Aβ oligomers (the band at 20 kDa) in CL2006 **(B)** and CL4176 **(C)** worms fed either vehicle or SVHRP using ImageJ software. Data are expressed as mean density of the indicated band based from three independent experiments. Error bars represent SD. ^∗^*p* < 0.05.

Since the paralysis of CL4176 is caused by the Aβ toxicity, naturally we wanted to examine if the effect of SVHRP on delayed paralysis was associated with a decrease in Aβ accumulation in transgenic CL4176. We analyzed Aβ species from the transgenic *C. elegans* fed with or without SVHRP by Western blotting using antibodies against Aβ (6E10). Another *C. elegans* strain, CL2006, constitutively expressing cytoplasmic human Aβ_1-42_ in the body wall muscle cells, was considered too. This strain displays an age-related progressive reduction of muscle specific motility which is related to the accumulation of both Aβ_1-42_ fibrils and oligomers. **Figure 2A** shows Aβ-immunoreactive (6E10) bands detected in the tissues from the transgenic worms (CL2006, CL4176, and the control strain CL802) fed with or without SVHRP. The visual observation of immunoblot showed a specific reduction of Aβ oligomeric species, which are responsible for the toxicity and initiation of synaptic dysfunction observed in AD (Lesne et al., 2006; Oddo et al., 2006; Nimmrich and Ebert, 2009; Taylor et al., 2010). The mean densities of the Aβ oligomer bands at 20 kDa were analyzed. Statistically, SVHRP significantly reduced the oligomers both in CL4176 and in CL2006 (**Figures 2B,C**; *n* = 3; CL2006, Ctrl vs. SVHRP, *p* = 0.01; CL4176, Ctrl vs. SVHRP, *p* = 0.013).

### SVHRP Reduces Amyloid Deposition in Transgenic *C. elegans*

**FIGURE 3:**
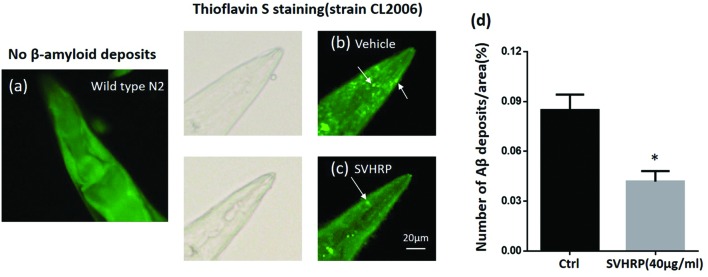
**Aβ deposits in transgenic *C. elegans* CL2006 fed with or without SVHRP.** Representative images of *C. elegans* with thioflavin S staining in the wild type **(a)**, or in the transgenic strain CL2006 fed with **(c)** or without SVHRP **(b)**. The number of deposits (arrows) was scored in the worm head **(d).** Data were obtained from three experiments with 24 worms in each group (*n* = 72). The quantity is expressed as mean number of β-amyloid deposits in the head region /anterior area of the worm. Error bars indicate SE. ^∗^*p* < 0.05.

Further, we scored the number of amyloid-reactive deposits in the head of *C. elegans* strain CL2006 to evaluate whether the inhibitory effect of SVHRP on Aβ oligomerization would reduce the degree of amyloidosis. **Figures 3b,c** shows Aβ deposits (white arrows) detected in CL2006 but not the wild type (N2) (**Figure 3a**), as previously observed (Link, 1995). **Figure 3d** shows that the mean number of Aβ deposits was significantly reduced in worms fed with 40 μg/ml SVHRP. These results implied that the protective effect of SVHRP against Aβ induced toxicity in the worms, may be due to inhibit Aβ oligomerization and deposition.

### SVHRP Inhibits Superoxide Production in *C. elegans*

**FIGURE 4:**
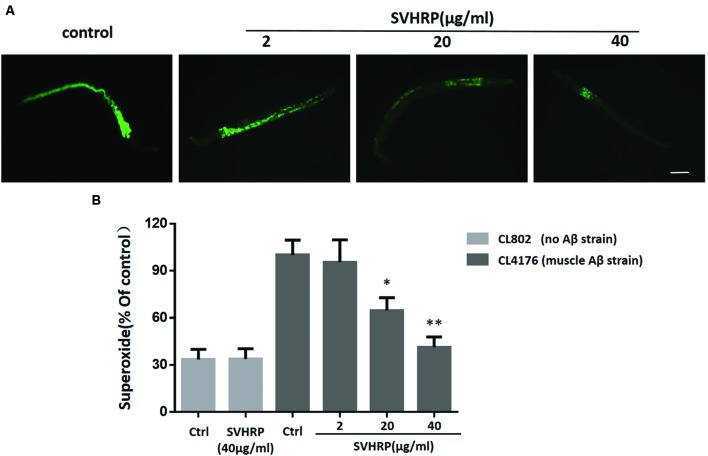
**Effect of SVHRP on reactive oxygen species (ROS) production in transgenic *C. elegans* strains.** Age-synchronized CL4176 or CL802 worms, fed vehicle or different concentrations of SVHRP (2, 20, 40 μg/ml) were collected 36 h after the temperature rise followed by the DCF (2, 7-dichlorofluorescein diacetate) assay for ROS described in Materials and Methods. **(A)** DCF fluorescent images of nematodes fed vehicle or different concentrations of SVHRP. Scale bars: 100 μm. **(B)** The DCF fluorescence intensity was detected by a microplate reader at 485 nm excitation and 530 nm emission. Results are expressed as percentage of fluorescence (%DCF) relative to vehicle-treated controls, which is set as 100%. Data were obtained from three experiments with 50 worms in each experiment. Error bars indicate SE, ^∗^*p* < 0.05, ^∗∗^*p* < 0.01 vs. control group).

Oxidative stress occurs in transgenic *C. elegans* strains expressing human Aβ_1-42_ and the increase of superoxide levels precedes the paralysis (Gutierrez-Zepeda et al., 2005; Luo, 2006). Given that a number of studies have associated oxidative stress with Aβ toxicity and AD, we investigated the *in vivo* ROS activity in transgenic strain, CL4176, to determine whether SVHRP has effect in reducing ROS in transgenic *C. elegans.* Three concentrations of SVHRP (2, 20, and 40 μg/ml) were added to the transgenic *C. elegans* from egg until the end of the temperature upshift. Compared to the control strain CL802, ROS levels rose significantly in Aβ expressing transgenic strains (**Figure 4B**). As expected, feeding SVHRP attenuated the intracellular levels of ROS in a dose-dependent manner (**Figures 4A,B**). Feeding 40 μg/ml SVHRP exhibited most significant reduction (Ctrl, 100 ± 16.5%; SVHRP, 41.4 ± 11.2%; n = 3; *p* = 0.007). These results suggest that the delayed onset of Aβ related paralysis phenotype in CL4176 worms with SVHRP treatment might be, in part, due to the ROS suppressing properties of SVHRP.

### SVHRP Suppresses Neuronal Aβ Expression-Induced Defect in Chemotaxis Behavior

**FIGURE 5:**
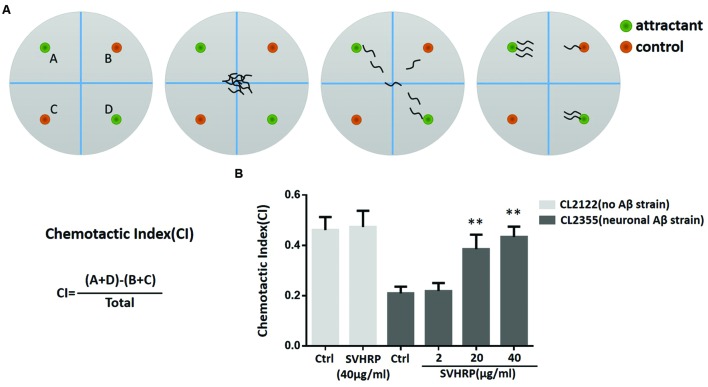
**Assays for chemotaxis behavior in neuronal Aβ-expressing strain CL2355. (A)** Schematic diagram of the chemotaxis assay. Plates were divided into quadrants two test (A&D) and two controls (B&C). Sodium azide was also included with the attractant and control odorant to paralyze worms. Worms were placed at the center of the plate and after 60 min worms were counted on each quadrant. Schematic examples of neutral and attractant are indicated. **(B)** The Chemotactic Index (CI) in the neuronal strain CL2355 and the transgenic control strain CL2122 fed with vehicle or different concentration of SVHRP (2, 20, 40 μg/ml). Data were obtained from three experiments with 60 worms in each group. Error bars indicate SD (^∗∗^*p* < 0.01 vs. CL2355 control group).

Numerous studies have suggested that small oligomeric forms of Aβ-peptide are the main toxic species to neurons. Next, we used a transgenic strain, CL2355, which Aβ was expressed in the neuronal cells, to investigate whether the inhibitory effect of SVHRP on Aβ oligomerization would protect neuron against Aβ-induced toxicity. The neuronal controlled behaviors, chemotaxis, were assayed in these worms. The CI is a measure of the fraction of worms that are able to arrive at the location of the attractants (Adachi et al., 2008; Margie et al., 2013). **Figure 5B** shows that the CI value was significantly reduced in transgenic strain CL2355 compared to the control strain CL2122 (Ctrl CI_CL2355_, 0.21 ± 0.03 vs. Ctrl CI_CL2122_, 0.46 ± 0.05; *n* = 3; *p* < 0.001). SVHRP(40 μg/ml) feeding of the control strain had no effect on their CI (Ctrl CI_CL2122_, 0.46 ± 0.05, vs. SVHRP CI_CL2122_, 0.47 ± 0.06; *n* = 3; *p* = 0.78), but it obviously normalized the reduced CI in neuronal Aβ transgenic strain in a concentration-dependent manner (0.21 ± 0.03 for Ctrl CL2355 worms and 0.22 ± 0.03, 0.39 ± 0.06, 0.43 ± 0.04 for nematodes fed 2, 20, 40 μg/ml SVHRP). These results suggest that SVHRP might be able to protect neuro against Aβ-induced toxicity and rescue neuronal Aβ expression-induced defects in chemotaxis behavior.

## Discussion

Here, we first observe the effects of SVHRP-polypeptide extract from scorpion venoms in Aβ transgenic *C. elegans*. By using transgenic *C. elegans*, CL4176, we examined if SVHRP could reduce paralysis resulting from Aβ_1-42_ peptide expression. Our data showed a significant delay of Aβ induced paralysis in SVHRP-treated (20, 40 μg/ml) worms (**Figure 1B**) and the effects were dose-dependent. The lower dose of SVHRP (2μg/ml) was too low to produce protective effects and the higher doses (80 μg/ml) tended to be toxic, causing the death of worms. A recent study evaluated the drug metabolism rate in *C. elegans* (Zheng et al., 2013) and they found the metabolism rate increased gradually with the passing of time and reached the highest at the time interval of 8 to 12 h in *C. elegans* (Zheng et al., 2013). In this study, feeding the CL4176 worms with SVHRP before Aβ expression, or after Aβ expression by temperature upshift could not delay the paralysis (data not shown) (see diagram in **Figure 1A**) and similar results were obtained in worms fed the EGb 761 (Wu et al., 2006) and Oleuropein Aglycone (Diomede et al., 2013), suggesting that short duration of feeding may not be sufficient to alleviate Aβ toxicity.

Substantial studies implicate intracellular Aβ oligomers in early events are related to AD (Kienlen-Campard et al., 2002; Umeda et al., 2015) and responsible for the early cognitive impairment observed in patients before the amyloid plaque deposits appear (Kayed et al., 2003; Nimmrich and Ebert, 2009; Taylor et al., 2010). Accordingly, inhibition of Aβ aggregation would be a potential therapeutic strategy. Some oligomers (20 kDa) were inhibited by SVHRP as observed in this study (**Figure 2A**). A similar effects have previously been reported for a cocoa peptide (Martorell et al., 2013) and glycitein from soybeans (Gutierrez-Zepeda et al., 2005). Moreover, the inhibitory effects of SVHRP on Aβ oligomerization would affect amyloid deposition. The analysis of fibrillar deposits in adult CL2006 worms showed a significantly reduction in SVHRP-fed worms (**Figure 3D**). These results would suggest that the paralysis suppression by SVHRP might be mediated by inhibiting Aβ oligomerization and amyloid deposition, which is directly associated with Aβ-toxicity in the *C. elegans* transgenic model. Recently, some studies have found Aβ has some physiological roles on synaptic plasticity and memory (Morley et al., 2010; Puzzo et al., 2011). Since the absence of endogenous Aβ production in the wild type worms, more research is needed to investigate the impact of SVHRP on the physiological production of Aβ and synaptic plasticity.

According to the “amyloid cascade” hypothesis, oxidative stress induced by Aβ may be involved in the pathogenesis of AD (Butterfield, 1997). In this study, we report SVHRP reduced superoxide production in the transgenic *C. elegans* strains CL4176. In *C. elegans*, ROS production is a typical outcome of amyloid deposition and the effect of SVHRP on ROS production may be a consequence of amyloid reduction. But we can’t exclude the possibility that SVHRP has direct scavenging effects on ROS, since the antioxidant properties of SVHRP have also been observed in the experimental model where increased oxidative stress was not induced by Aβ (Yin et al., 2014). A large number of studies in AD mouse model have suggested the antioxidant-based therapies as a potential avenue to mitigate AD (Moreira et al., 2006; Dumont et al., 2009) and the antioxidant activity of SVHRP might contribute to protecting against the Aβ-induced damage.

The transgenic *C. elegans* strains, CL4176 and CL2006, which express the human Aβ_1-42_ in muscle tissues, reflect the toxicity of Aβ oligomers to muscle cells. However, in AD, Aβ is primarily toxic to neurons. We chose another transgenic strain, CL2355, expressing Aβ in neuronal cells to investigate whether SVHRP could protect neuron against Aβ-induced toxicity in *C. elegans.* These nematodes exhibit defects in chemotaxis behavior due to Aβ induced toxicity (Wu et al., 2006). By using the transgenic strain CL2355, we found SVHRP could also protect neuronal cells against amyloid toxicity *in vivo* results in a strong protection against the pathological phenotype (**Figure 5B**). Our previous experiments have demonstrated that SVHRP had neurotrophic and neuroprotective effects (Wang et al., 2014; Yin et al., 2014). SVHRP could enhance neurogenesis and stimulate neural maturation by up-regulating BDNF (Wang et al., 2014). However, the neurotrophic effects of SVHRP by up-regulating BDNF may not contribute to rescuing Aβ toxicity induced defects in chemotaxis behavior, since *C. elegans* really do not have BDNF *per se.* Further research using higher order experimental models is needed to clarify the exact molecular and cellular mechanisms underlying SVHRP neuroprotective activity.

In summary, in this study, we found that SVHRP-polypeptide extract from scorpion venoms alleviated Aβ-induced paralysis and chemotaxis dysfunction in the transgenic *C. elegans* expressing Aβ. SVHRP also modulated Aβ oligomers and attenuated levels of ROS in the transgenic *C. elegans*. Though the mechanisms behind the protective effect of SVHRP in *C. elegans* remain unclear, our results suggest SVHRP is worthy of further studies with murine models in order to validate the effectiveness of this peptide.

## Author Contributions

Conceived and designed the experiments: X-GZ, XW, SL, JZ. Performed the experiments: X-GZ, XW. Analyzed the data: X-GZ, XW, T-TZ. Contributed reagents/Materials/Analysis tools: X-FW, YP, W-QZ, SL, JZ. Wrote the paper: X-GZ, XW.

## Conflict of Interest Statement

The authors declare that the research was conducted in the absence of any commercial or financial relationships that could be construed as a potential conflict of interest.
